# Association of blood lead concentrations with mortality in older women: a prospective cohort study

**DOI:** 10.1186/1476-069X-8-15

**Published:** 2009-04-03

**Authors:** Naila Khalil, John W Wilson, Evelyn O Talbott, Lisa A Morrow, Marc C Hochberg, Teresa A Hillier, Susan B Muldoon, Steven R Cummings, Jane A Cauley

**Affiliations:** 1 University of Pittsburgh, Department of Epidemiology, Pittsburgh, PA, USA; 2 University of Pittsburgh, Department of Biostatistics, Pittsburgh, PA, USA; 3 University of Pittsburgh, Department of Psychiatry, Pittsburgh, PA, USA; 4 University of Maryland, Departments of Medicine, Epidemiology, and Preventive Medicine Baltimore, MD, USA; 5 Center for Health Research Northwest/Hawaii, Kaiser Permanente, Portland, OR, USA; 6 University of Louisville, School of Public Health and Information Sciences, Louisville, KY, USA; 7 California Pacific Medical Center Research Institute, San Francisco Coordinating Center, San Francisco, CA, USA

## Abstract

**Background:**

Blood lead concentrations have been associated with increased risk of cardiovascular, cancer, and all-cause mortality in adults in general population and occupational cohorts. We aimed to determine the association between blood lead, all cause and cause specific mortality in elderly, community residing women.

**Methods:**

Prospective cohort study of 533 women aged 65–87 years enrolled in the Study of Osteoporotic Fractures at 2 US research centers (Baltimore, MD; Monongahela Valley, PA) from 1986–1988. Blood lead concentrations were determined by atomic absorption spectrometry. Using blood lead concentration categorized as < 8 μg/dL (0.384 μmol/L), and ≥ 8 μg/dL (0.384 μmol/L), we determined the relative risk of mortality from all cause, and cause-specific mortality, through Cox proportional hazards regression analysis.

**Results:**

Mean blood lead concentration was 5.3 ± 2.3 μg/dL (range 1–21) [0.25 ± 0.11 μmol/L (range 0.05–1.008)]. After 12.0 ± 3 years of > 95% complete follow-up, 123 (23%) women who died had slightly higher mean (± SD) blood lead 5.56 (± 3) μg/dL [0.27(± 0.14) μmol/L] than survivors: 5.17(± 2.0) [0.25(± 0.1) μmol/L] (*p *= 0.09). Women with blood lead concentrations ≥ 8 μg/dL (0.384 μmol/L), had 59% increased risk of multivariate adjusted all cause mortality (Hazard Ratio [HR], 1.59; 95% confidence interval [CI], 1.02–2.49) (p = 0.041) especially coronary heart disease (CHD) mortality (HR = 3.08 [CI], (1.23–7.70)(p = 0.016), compared to women with blood lead concentrations < 8 μg/dL(< 0.384 μmol/L). There was no association of blood lead with stroke, cancer, or non cardiovascular deaths.

**Conclusion:**

Women with blood lead concentrations of ≥ 8 μg/dL (0.384 μmol/L), experienced increased mortality, in particular from CHD as compared to those with lower blood lead concentrations.

## Background

Lead is a multitargeted toxicant, affecting cardiovascular, renal and nervous systems, and may contribute to morbidity and mortality through its adverse impacts on these systems [1,2].

An association between lead and mortality has been observed in both occupational and community based cohorts [3]. Results from the second National Health and Nutrition Examination Survey (NHANES II, 1976–1980) showed that blood lead concentration was an important predictor of mortality [4]. Individuals with baseline blood lead concentrations of 20 to 29 μg/dL [0.96 to 1.39 μmol/L (To convert μg/dL of blood lead into μmol/L, multiply by 0.048)] experienced 46% increased all cause mortality, relative to those with blood lead concentrations less than 10 μg/dL(0.48 μmol/L) [5]. In NHANES III (1988–1994) an increased risk of death from all causes, cardiovascular disease, and cancer was associated with much lower blood lead concentrations of 5–9 μg/dL(0.24–0.43 μmol/L) as compared to those with < 5.0 μg/dL [6]. Furthermore Menke et al documented a 25% increased all cause and 55% increased cardiovascular mortality in NHANES III (1988–1994) at considerably lower blood lead concentrations: > 3.62 μg/dL (0.17 μmol/L) as compared to those with < 1.94 μg/dL (0.09 μmol/L) [7]. However they did not observe an association between blood lead and cancer mortality in this range of exposure.

Environmental exposures to lead have been associated with hypertension and the incidence of clinical cardiovascular endpoints such as coronary heart disease, stroke, and peripheral artery disease [8]. Cardiac abnormalities such as left ventricular hypertrophy [9] and alteration in cardiac rhythm [10] have also been documented with lead exposure. Higher blood concentrations have been associated with cognitive and neuromuscular decline [11-13], and renal effects [2,14-16] all of which could contribute to an increased risk of mortality. The effects of blood lead concentrations on cancer mortality however, are poorly understood. In the current analysis we prospectively examined the association of blood lead concentrations and mortality in a cohort of 533 white women with mean age of 72.5 (± 4.4) (range: 68–89) years and mean blood lead concentrations of 5.3 μg/dL (± 2.3 SD) (range: 1–21). We hypothesized that woman with blood lead concentrations above a threshold will experience higher total and cause specific mortality.

## Methods

### Study Population

The Study of Osteoporotic Fractures (SOF) is a longitudinal cohort study that enrolled 9704 white women from 1986 to 1988 using population-based listings in Baltimore, MD; Minneapolis, MN; Portland, OR; and the Monongahela Valley near Pittsburgh, PA. To be eligible to participate, women had to be aged 65 years or older and ambulatory. The lead ancillary study was conducted in 1990–1991 in 533 white women aged 65–87 years enrolled in SOF at either the University of Pittsburgh or University of Maryland clinics. The participants in this study of blood lead concentrations represent a convenience sample obtained from two of the clinical centers of the Study of Osteoporotic Fractures.

Initially, we examined the correlates of blood lead and the association of blood lead concentrations to cognitive function [17]. Analyses were performed by categorizing the study participants into three groups corresponding to the upper and lower 15^th ^percentiles of the distribution of blood lead. Thus, the three groups were: low [= 3 μg/dl (0.14 μmol/L), lower 15^th ^percentile; referent; n = 122)]; medium [4–7 μg/dl (0.19–0.34 μmol/L); n = 332]; and high [= 8 μg/dl (0.38 μmol/L), upper 15^th ^percentile; n = 79]. This categorization was determined a priori [17]. We found a relationship between blood lead concentration as low as 8 μg/dL and worse cognitive function as measured by the part B of Trailmaking Test, but this association was confined to the rural SOF clinic [17,18]. In a recent analysis in the same population, we found a significantly higher risk of falls and fractures in older women at blood lead concentration ≥ 4 μg/dL (0.19 μmol/L), and faster rate of bone loss at ≥ 8 μg/dL (0.38 μmol/L), when compared to women with blood lead concentration < 3 μg/dL (0.14 μmol/L) [19]. In the current paper, we extend the lead study to mortality outcomes. The protocol and consent forms were approved by the institutional review boards at the participating institutions. All women provided written informed consent.

### Questionnaire and interview

Each participant completed a baseline questionnaire that ascertained her education and health behaviors including smoking, alcohol use, and walking for exercise. They also were asked about physician diagnosed diabetes, hypertension (measured blood pressure > 160/90 or thiazide use). They were asked about current use of estrogen.

### Examinations

At the baseline clinic examination, each participant had her blood pressure measured by manual mercury sphygmomanometer. BMD of total hip was measured at the second (1988–1990) examination by Dual energy X-ray absorptiometry (DXA) using Hologic QDR 1000 scanners (Bedford, Mass). Height and weight were obtained using a Harpenden stadiometer (Holtain Ltd, Crymych, UK) and a standard balance beam, respectively, and body mass index (BMI) was calculated as weight divided by height squared (Kg/m^2^).

### Mortality

The methods of determining deaths in SOF have been published[20,21]. Briefly, participants were contacted every 4 months by postcard after visit 2 (1991–92) over 12 (± 3) years of follow-up. These contacts are > 95% complete. Deaths were confirmed by death certificates. Hospital discharge summaries were obtained for 41(33%) of deceased participants. The underlying cause of death was coded by a clinical epidemiologist using the *International Classification of Diseases, Ninth Revision, Clinical Modification*, and categorized as due to cardiovascular disease (CVD) including all diseases of circulatory system except those involving veins and lymphatics [ICD-9-CM codes 425, 429.2, 440–444, 428, 401–404, 410–414, 430–438, and 798.]; Coronary heart disease (CHD) [ICD-9-CM 410–414]; Stroke [ICD-9-CM 430–438]; cancer [ICD-9-CM codes 140 to 239] and all other deaths.

### Blood Lead Measurements

A 5.0 ml sample of whole blood was drawn into Vacutainer tubes (BD Vacutainer Systems, Rutherford, New Jersey). Blood samples were analyzed at the Clinical Chemistry Laboratory of the University of Maryland, certified for the analysis of lead in blood by the Occupational Safety and Health Administration and Centers for Disease Control and Prevention, and documents a lower limit of detection for lead of 1 μg/dL. Blood lead concentrations were determined by graphite furnace atomic absorption spectrometry (AAS model 5100, HGA with Zeeman Effect background correction: Perkin Elmer, Norwalk, Connecticut). To determine intralaboratory measurement variability in lead concentration and the stability of samples over 1 year, 100 samples (50 from each clinic) were drawn from randomly selected women during a clinic visit, one year later. The intraclass correlation coefficient for the duplicates was 0.88. Mean values of 4.76 μg/dL (range, 1–13 μg/dL) [0.23(range 0.05–0.62 μmol/L)], and 4.67 μg/dL (range, 1–12 μg/dL) [0.22 μmol/L (range 0.05–0.58) were obtained for the first and second determinations, respectively[17]

### Statistical Analysis

Preliminary analyses were performed by categorizing the study participants into three groups corresponding to the upper and lower 15^th ^percentiles of the distribution of blood lead. Thus, the three groups were: low [≤ 3 μg/dl (0.14 μmol/L), lower 15^th ^percentile; referent; n = 122)]; medium [4–7 μg/dl (0.19–0.34 μmol/L); n = 332]; and high [≥ 8 μg/dl (0.384 μmol/L), upper 15^th ^percentile; n = 79]. This categorization was determined a priori based on our previous study of blood lead and cognitive functions [17]. Analysis suggested that mortality outcome was only significant at the highest 15^th ^percentile i.e., ≥ 8 μg/dL (0.384 μmol/L) of blood lead, when compared to the referent and the medium group, we therefore combined these two categories into a new reference group with lead level < 8 ug/dl (0.384 μmol/L). Furthermore, preliminary analysis by dividing the participants into quintiles, also suggested that only the top quintile (80^th ^percentile i.e., ≥ 8 μg/dL (0.384 μmol/L) of blood lead) showed elevated risk of death. We therefore dichotomized lead concentrations above and below 8 μg/dL (80^th ^and 85^th ^percentile was same i.e., ≥ 8 μg/dL(0.384 μmol/L) of blood lead), thus the two groups were: (< 8 μg/dL, referent, n = 454), and (≥ 8 μg/dL, n = 79).

We compared baseline characteristics by lead and mortality status, using chi-square tests for categorical variables and t-tests for continuous variables. Two-tailed p-values were used for all tests, at 5% statistical significance. Separate models were analyzed for all cause and cause specific mortality. CVD mortality was categorized into two subgroups: deaths due to stroke, and coronary heart disease.

We used Cox proportional hazards regression analysis to estimate the Hazard Ratio (HR) and 95% confidence intervals (CI) to determine association between blood lead concentration and mortality. As done previously in SOF[22] we assessed variables for inclusion in the models based on biological plausibility and documented association in literature with blood lead [4,5] and mortality [23]. Selected on these criteria, we controlled for the following variables into all models: age increase per 5 years, clinic, BMI, education, smoking, alcohol intake, estrogen use, hypertension, total hip BMD, walking for exercise, and diabetes.

The proportionality assumptions of the Cox models were confirmed with Schoenfeld residuals. We plotted cumulative survival in two blood lead concentrations groups over follow-up period by Kaplan- Meier curves. Data were analyzed with Stata (edition 9, StataCorp, College Station, Texas).

## Results

The women in lead ancillary study were compared to the rest of SOF study participants. Lead study cohort was younger in age from the rest of the SOF participants (Table 1). A lower proportion of women in lead cohort was hypertensive, or walked for exercise. Proportion of diabetics was higher in the lead study. However, BMI, education, alcohol use, smoking, use of estrogen, bone density in total hip were comparable.

**Table 1:** Comparison of participants' baseline characteristics between SOF and lead ancillary study.

Characteristics (N total = 9704)	Lead cohort N = 533	SOF participants N = 9171	P value
Age (years), mean SD	70 ± 4	72 ± 5	< 0.001
Body mass index (kg/m^2)^), mean SD	27 ± 5	26 ± 4	0.147
Education (years), mean SD	12 ± 3	13 ± 3	0.135
Alcohol (drinks/wk), mean SD	1.9 ± 4	1.8 ± 4	0.737
Current smoker, n (%)	65 (12)	902 (10)	0.079
Hypertension, n (%)	153 (29)	3594 (39)	< 0.001
Diabetes, n (%)	55 (10)	652 (7)	0.006
Current estrogen use, n (%)	67 (13)	1400 (15)	0.091
Walk for exercise (yes/no), n (%)	213 (40)	4653 (51)	< 0.001
Total hip, bone density (g/cm^2^), mean SD	0.77 ± 0.13	0.76 ± 0.13	0.536

Mean blood lead concentration was 5.3 ± 2.3 μg/dL (range 1–21) [0.25 ± 0.11 μmol/L (range 0.05–1.008)]. A total of 123 (23%) women died over a mean follow up of 12.0 (± 3.0) years. Women with ≥ 8 μg/dL(0.384 μmol/L), blood lead concentration had higher alcohol intake, were more likely to smoke, and had 8% lower total hip BMD (Table 2).

**Table 2:** Baseline characteristics in women in SOF lead ancillary study by blood lead concentrations

Characteristics by blood lead concentration	< 8 μg/dL (< 0.384 μmol/L) N = 453	≥ 8 μg/dL (≥ 0.384 μmol/L) N = 79	P value
All cause mortality, n (col %)	96 (21)	27 (34)	0.011
Age (years), mean ± SD	70 ± 4	70 ± 5	0.196
Body mass index (kg/m^2)^), mean ± SD	27 ± 5	26 ± 4	0.075
Education (years), mean ± SD	12 ± 3	13 ± 3	0.415
Alcohol (drinks/wk), mean ± SD	1.5 ± 3	2.9 ± 5	0.003
Current smoker, n (%)	46 (10)	20 (25)	0.001
Hypertension, n (%)	128 (28)	25 (32)	0.539
Diabetes, n (%)	48 (11)	7 (9)	0.644
Current estrogen use, n (%)	60 (13)	7 (9)	0.281
Walk for exercise (yes/no), n (%)	183 (40)	30 (38)	0.696
Total hip, bone density (g/cm^2^), mean ± SD	0.77 ± 0.13	0.72 ± 0.12	< 0.006

**Figure 1:**
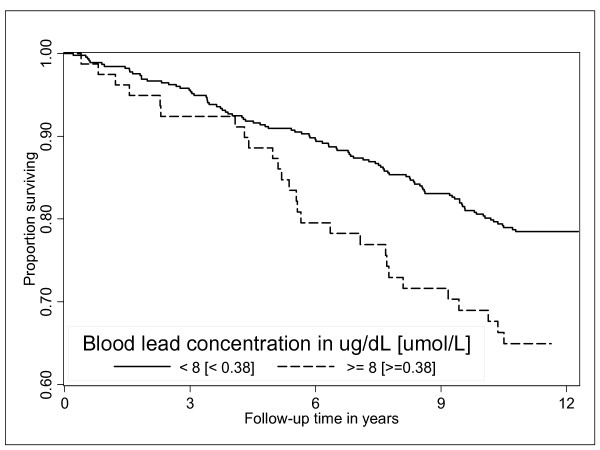
**Cumulative survival with blood lead concentrations < 8 μg/dL (< 0.384 μmol/L), and ≥ 8 μg/dL (≥ 0.384 μmol/L) in women in SOF**. Cumulative survival associated with blood lead concentrations < 8 μg/dL (< 0.384 μmol/L), and ≥ 8 μg/dL (≥ 0.384 μmol/L), in women in SOF lead ancillary study (Log rank test P < 0.007)

Women who died had 7% higher mean (± SD) blood lead concentration 5.6 (3) μg/dL, [0.27(± 0.14) μmol/L] than survivors: 5.17(± 2.0) [0.25(± 0.1) μmol/L] μg/dL (*p *= 0.09, Table 3). As compared to survivors, women who died were older, more likely to smoke and to have hypertension. A lower proportion of women who died reported walking for exercise. Compared to women whose blood lead concentrations were < 8 μg/dL, (< 0.384 μmol/L) survival decreased more rapidly in women with blood lead concentration ≥ 8 μg/dL (≥ 0.384 μmol/L) (Figure 1). Age, clinic, smoking, hypertension, and total hip BMD were significantly associated with mortality in women with blood lead concentration ≥ 8 μg/dL (≥ 0.384 μmol/L) (Table 4).

**Table 3:** Baseline characteristics in women in SOF lead ancillary study by survival/mortality status.

Characteristic	Died N = 123	Survived N = 410	P value
Age (years), mean ± SD	72 ± 5	70 ± 4	0.001
Education (years), mean ± SD	12 ± 3	12 ± 3	0.811
Body mass index(kg/m^2)^), mean ± SD	26 ± 5	27 ± 5	0.164
Alcohol (drinks/wk), mean ± SD	1.9 ± 5	1.8 ± 4	0.840
Current smoker, n (%)	25 (20)	41(10)	0.002
Hypertension, n (%)	47 (39)	106 (26)	0.007
Diabetes, n (%)	15 (12)	40 (10)	0.435
Current estrogen use, n (%)	12 (10)	55 (13)	0.283
Walk for exercise (yes/no), n (%)	41 (33)	172 (42)	0.087
Total hip, bone density (g/cm^2^), mean ± SD	0.71 ± 0.13	0.77 ± 0.13	< 0.001
All cause death, B-Pb (μg/dL) [μmol/L], mean ± SD	5.56 ± 3 [0.27 ± 0.14]	5.17 ± 2 [0.25 ± 0.1]	0.093
CVD death, B-Pb (μg/dL), [μmol/L], mean ± SD	5.81 ± 3 [0.28 ± 0.14 ]	5.19 ± 3 [0.25 ± 0.14]	0.059
CHD death, B-Pb (μg/dL), [μmol/L], mean ± SD	5.61 ± 2 [0.27 ± 0.1]	5.19 ± 2 [0.25 ± 0.1]	0.373
Stroke death, B-Pb (μg/dL), [μmol/L], mean ± SD	6.33 ± 3 [0.30 ± 0.14]	5.21 ± 2 [0.25 ± 0.1]	0.028
Cancer death, B-Pb (μg/dL), [μmol/L], mean ± SD	5.34 ± 2 [0.26 ± 0.1]	5.25 ± 2 [0.25 ± 0.1]	0.812
Other death, B-Pb (μg/dL), [μmol/L], mean ± SD	5.39 ± 4 [0.26 ± 0.19]	5.25 ± 2 [0.25 ± 0.1]	0.745

*B-Pb: blood lead concentration

**Table 4:** Multivariable model of all cause mortality by blood lead concentrations

Variable name	Hazard Ratio	95% confidence interval	p-value
Lead ≥ 8 μg/dL (≥ 0.384 μmol/L)	1.78	1.16, 2.73	0.008
Age increase per 5 years	1.62	1.36, 1.92	< 0.001
Clinic	1.56	1.06, 2.30	0.024
Body mass index(kg/m^2)^)	0.98	0.94, 1.02	0.239
Education (years)	0.98	0.92, 1.05	0.620
Alcohol (drinks/wk)	0.99	0.95, 1.04	0.814
Current smoker	1.84	1.18, 2.88	0.008
Hypertension	1.73	1.20, 2.49	0.003
Diabetes	1.11	0.83, 1.49	0.468
Current estrogen use	0.75	0.42, 1.38	0.363
Walk for exercise	0.70	0.48, 1.02	0.066
Total hip, bone density (g/cm^2^)	0.05	0.01, 0.19	0.001

**Figure 2:**
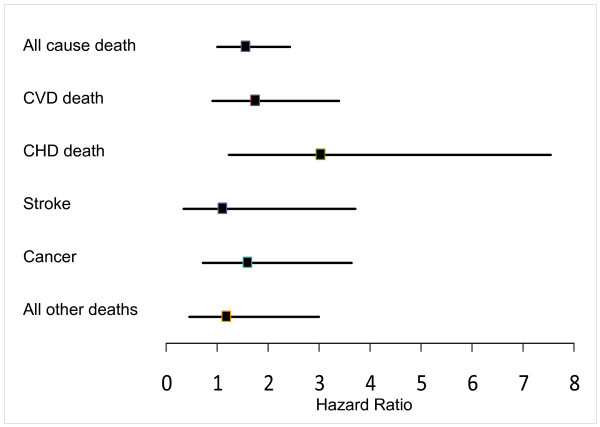
**Adjusted hazards ratios and 95% confidence interval of mortality in SOF participants with blood lead concentrations ≥ 8 μg/dL (≥ 0.384 μmol/L)**. Adjusted hazard ratios and 95% confidence interval of all cause mortality and cause specific mortality in SOF lead ancillary study participants with blood lead concentrations ≥ 8 μg/dL (≥ 0.384 μmol/L), compared to referent < 8 μg/dL (< 0.384 μmol/L),

Women with baseline blood lead concentration of ≥ 8 μg/dL (≥ 0.384 μmol/L) had a 73% increased risk of dying. (Age and clinic adjusted hazard ratio [HR], 1.73; 95% confidence interval [CI], 1.12–2.68) (p = 0.014) compared to women in blood lead < 8 μg/dL (0.38 μmol/L). With further adjustment for covariates women with ≥ 8 μg/dL (0.384 μmol/L) still had 59% higher risk of all cause mortality (HR = 1.59; 1.02–2.49) (p = 0.041), compared to women with < 8 μg/dL (0.38 μmol/L) of blood lead. Although the multivariate adjusted hazards ratio (95% CI) for CVD mortality for women who had ≥ 8 μg/dL (≥ 0.384 μmol/L) versus the < 8 μg/dL (< 0.38 μmol/L) blood lead concentrations was not significant; 1.78(95% CI, 0.92–3.45), p = 0.089, women in higher lead group experienced 3 fold higher risk of mortality due to coronary heart disease 3.08(95% CI, 1.23–7.70), p < 0.016. There was no association of blood lead and mortality from stroke, cancer and other causes (Table 5 and Figure 2).

**Table 5:** Hazards Ratio (HR) and 95% confidence interval of all cause mortality by blood lead concentrations

Cause of death	Deaths	Blood lead concentration (μg/dL) [μmol/L]		*P*_*value*_
		(< 8) [< 0.384 ]	(≥ 8) [≥ 0.384 ]	
All cause death, n (Col %)	123	96 (21%)	27 (34%)	0.018*
Age, clinic adjusted		1.0	1.73 (1.12, 2.68)	0.014
Multivariate adjusted ^a^		1.0	1.59 (1.02, 2.49)	0.041
Cardiovascular disease ^b^, n(%)	54	41(9)	13 (16)	0.044*
Age, clinic adjusted		1.0	1.90 (1.00, 3.63)	0.054
Multivariate adjusted ^c^		1.0	1.78 (0.92, 3.45)	0.089
Coronary heart disease ^d^, n (%)	23	15 (4)	8 (11)	0.006*
Age, clinic adjusted		1.0	3.54 (1.48, 8.45)	0.004
Multivariate adjusted ^e^		1.0	3.08 (1.23, 7.70)	0.016
Stroke ^f^, n(%)	21	17 (4)	4 (5)	0.578*
Age, clinic adjusted		1.0	1.16 (0.34, 4.00)	0.816
Multivariate adjusted ^g^		1.0	1.13 (0.34, 3.81)	0.840
Cancer ^h^, n(%)	38	30 (7)	8 (10)	0.262*
Age, clinic adjusted		1.0	1.70 (0.77, 3.75)	0.185
Multivariate adjusted ^i^		1.0	1.64 (0.73, 3.71)	0.231
All other deaths ^j^, n(%)	31	25 (7)	6 (10)	0.289*
Age, clinic adjusted		1.0	1.51 (0.61, 3.72)	0.370
Multivariate adjusted ^k^		1.0	1.22 (0.48, 3.10)	0.673

*Chi -square p-value only for percentage of deaths in two blood lead strata, the rest are hazards ratio p values.

^a,c,e,g,i,k ^The multivariate model included the following: age, clinic, BMI, education, smoking, alcohol intake, estrogen use, hypertension, walking for exercise, diabetes, and total hip BMD.

^b ^ICD9 Code: All deaths due to CVD, including all diseases of circulatory system except those involving veins and lymphatics: 425,429.2, 440–444,428,401–404,410–414,430–438, and 798.

^d ^ICD9 Code: deaths due to coronary heart disease: 410–414.

^f ^ICD9 Code: Deaths due to stroke: 430–438.

^h ^ICD9 Code: Deaths due to cancer: 140–239.

^j ^ICD9 Code: All other deaths: Non CVD and non cancer deaths

## Discussion

Blood lead was an important predictor of all cause mortality in this cohort of community dwelling older women. Mortality was significantly higher in women with blood lead concentrations ≥ 8 μg/dL as compared to those with lower blood lead concentrations. Our results are consistent with earlier studies based on occupational cohorts [3,24-26] and the general population: NHANES II and III [4-7]. To our knowledge, this is the first study to look at the association between blood lead concentrations and mortality in older women with median age of 71 years (range 65–87).

Despite declines in blood lead concentrations during the past 30 years, environmental lead exposure continues to be a public health concern [2]. The use of organic lead as gasoline additive, initiated during the 1920s, was phased out in the US since 1976. Once released as combustion exhaust, particulate lead persists in air, water, and soil. Mean blood lead concentrations in the U.S. population in the NHANES III Phase 2 collected during 1991 to 1994 ages 20 to 49 years were 2.1 μg/dL[1]. The mean blood lead concentration for ages > 1 year has declined further to 1.45 μg/dL(0.07 μmol/L) as reported from the most recent survey (NHANES IV, 2001–2002)[27].

The skeleton is repository for 95% of absorbed lead and can serve as an endogenous source for many years after exposure. Lead may be mobilized from skeleton during conditions of high bone turn over, such as pregnancy, lactation, menopause and aging [28]. A 25% adjusted increase in median blood lead concentration was reported for post menopausal women, compared to premenopausal women in NHANES II [29]. Furthermore in NHANES III, blood lead concentrations were highest in ages 70 years and older [3.4 μg/dL,(0.16 μmol/L) ] [27].

A multitargeted toxicant, lead effects cardiovascular, renal and nervous systems and may contribute to morbidity and mortality through its adverse impacts on these systems. The increased cardiovascular mortality risk may reflect an effect on sub-clinical risk factors for disease. The evidence for this association is supportive [27]. As part of NHANES III, an increased risk of peripheral arterial disease, hypertension and renal dysfunction was observed in populations with an average blood lead concentration of 2 μg/dL (0.10 μmol/L) [2,14,27,30]. For example, the odds ratio of diastolic hypertension was 8.1 comparing women with a blood lead concentration of 4.0–31.1 μg/dL (0.19–1.5 μmol/L) to women with lower blood lead concentrations of 0.5–1.6 μg/dL(0.02–0.08 μmol/L). Other analyses support an association between blood lead and renal function impairment [14,31,32], and increased blood pressure [33,34], a biologically plausible relationship [35].

Lead contributes to nephrotoxicity, even at blood concentration below 5 ug/dL (0.24 μmol/L) [32]. Increase in blood pressure and an association with renal damage have also been observed after lead exposure in rodent models [36,37]. Alterations in signal transduction that involve renal pathways (eg, angiotensin and vasopressin) were reported in rat models [38-40]. Other mechanisms by which lead may increase cardiovascular risk include effects on neuromuscular and neuro-humoral regulation of vascular function, alteration in sodium transport, and alterations in calcium regulation [7,11,27,30,41-47].

Blood lead was associated with almost three fold risk in coronary heart disease (CHD) mortality (HR = 3.08) in our study. Our results are consistent with findings in which *bone *lead, a more accurate biomarker of chronic lead exposure than blood lead was associated with ischemic heart disease mortality [48]. A one standard deviation increase in patella and tibia lead concentrations was associated with greater risk for ischemic heart disease (Hazard Ratio for patella lead = 1.29; 95% CI, 1.02–1.62).

The pathogenesis of CHD is multifactorial; lead may be one of the mediators by two causal pathways; i.e., mediation through higher blood pressure [8] and by atherogenic process [49]. Lead-related atherosclerosis could be explained by several mechanisms, impairment of renal function [32], induction of oxidative stress [50] and endothelial dysfunction [50].

Previous studies have linked lead as low as 3.62 μg/dL (0.17 μmol/L)with an increased risk of stroke mortality [7,8]. In our study, we found no significant association with stroke, although the mean lead concentration in participants who died due to stroke was 22% higher as compared to the rest of the cohort (6.33 ug/dL vs. 5.21 ug/dL, p < 0.028)(0.30 vs.0.25 μmol/L)perhaps reflecting the small number of stroke deaths in this cohort (Table 3).

Lead is a toxic metal and categorized as probably carcinogenic to humans (Group 2A IARC 2004) [51 Monograph]. Associations between occupational lead exposure and cancers of brain, stomach, kidney and lung have been reported [52-54]. However among non occupational cohorts, there has been inconsistent evidence of an association between blood lead and cancer [5]. Individuals with mean blood lead concentrations 10–19 μg/dL (0.48–0.91 μmol/L) in the NHANES II cohort (1976–1980) did not have increased risk of cancer mortality, when compared to those with blood lead concentrations < 10 μg/dL (0.48 μmol/L).

Our results are consistent with this observation as the median value in the "≥ 8 μg/dL" lead group was 9 μg/dL (0.43 μmol/L). A higher risk of cancer deaths was only observed in with blood lead concentration > 20 μg/dL(0.96 μmol/L) [4]. Similarly, individuals with blood lead concentrations ≥ 3.62 μg/dL (0.17 μmol/L) in the NHANES III (1988–1994) did not have increased risk of cancer mortality when compared to those with < 1.94 μg/dL(0.09 μmol/L) [7]. In contrast, another analysis from the same population survey reported 44% increased risk of cancer death at blood lead concentrations ≥ 5 μg/dL (0.24 μmol/L) when compared to those with < 5 μg/dL [6].

Bone loss accelerates after menopause and bone demineralization may release bone lead into circulation [28]. Inverse association has been reported between mortality, (predominantly stroke deaths) and bone density [55]. In our study women with ≥ 8 μg/dL (≥ 0.384 μmol/L) blood lead had 8% lower total hip bone mineral density at baseline as compared to women with lower lead concentrations. We also observed that women who died had 7% higher blood lead concentration than survivors. In another analysis in older women, each SD increase in BMD loss at the hip was associated with a 1.3-fold increase in total mortality, adjusted for age, baseline BMD, diabetes, hypertension, incident fractures, smoking, physical activity, health status, weight loss, and calcium use. In particular, hip BMD loss was associated with increased mortality from coronary heart disease (relative hazard [RH] = 1.3 per SD; 95% CI, 1.0–1.8) [56].

Alternatively, osteoporosis and atherosclerosis may result from elevated concentrations of homocysteine, an amino acid whose normal metabolism depends on folate and vitamin B_12 _as cofactors. Lead and homocysteine both are associated with cardiovascular disease and cognitive dysfunction[57]. In subjects 50–70 years of age, blood lead and homocysteine concentrations were correlated (Pearson's r = 0.27, p < 0.01), homocysteine concentration increased 0.35 μmol/L per 1.0 μg/dL (0.05 μmol/L) increase in blood lead (p < 0.01). Homocysteine is an example of plausible mechanism that may mediate the affect of lead on the cardiovascular [58] and central nervous systems [59]. Taken together, more research is clearly needed to further our understanding of the mechanism of lead toxicity and these multisystem outcomes.

Compared to the rest of SOF participants, the lead study cohort was of comparatively younger age, and had lower proportion with hypertension [60]. The proportion of women with type 2 diabetes was higher in the lead cohort, we adjusted for diabetes in secondary analysis and the association of lead, and mortality remained significant. The number ofwomen who are older and atrisk is growing. Cardiovascular disease is the leading cause of mortality worldwide [61] and in the United States [8]. It kills nearlyhalf a million women in the United States every year, more than the nextfivecauses of death combined and nearly twice as many as all forms of cancer, including breast cancer [62]. Environmental toxicants such as lead may explain part of the burden of CVD.

There are several strengths to our study: our follow up was more than 95% complete and we adjudicated all mortality events. We followed women for more than12 years after the blood lead measures were obtained. We controlled for a number of covariates and cardiovascular risk factors. However, this study has several limitations; participation was limited to older Caucasian women, and the findings may not apply to men or nonwhite women. We did not determine co-contaminants such as cadmium that might be associated with cardiovascular disease through its known effects on kidney function [63,64]. There are factors that differ by lead concentrations and we could not measure may have confounded our results. For example we had no measure of renal function, homocysteine or lipid concentrations and thus we could not examine whether these measures influenced the association between lead and mortality. We relied on death certificates and discharge summaries were only available for 33% of participants which may result in some misclassification of cause of death [65]. Use of death certificates may be problematic for assigning a single cause of death, especially among the oldest women who often have multiple medical problems.

## Conclusion

Our study extends the findings of higher mortality associated with blood lead concentrations from NHANES III surveys to community dwelling older women. An increased mortality risk, especially coronary heart disease was found at blood lead concentrations ≥ 8 μg/dL (0.384 μmol/L). Our results add to the existing evidence of adverse affects of lead on health as seen in an older cohort who experienced greater historic environmental lead exposure.

## Abbreviations

BMD: Bone mineral density; CHD: Coronary Heart Disease; CVD: Cardiovascular Disease; DXA: Dual energy X-ray absorptiometry; HR: Hazard Ratio; ICD: International Classification of Diseases, Ninth, revision, Clinical Modification; μg/dL: microgram per deciliter; NHANES: National Health and Nutritional Examination Survey; SOF: Study of Osteoporotic Fractures; VDR: vitamin D receptor gene.

## Competing interests

JAC has received research support from Merck & Company, Eli Lilly & Company, Pfizer Pharmaceuticals, and Novartis Pharmaceuticals. She has also received consulting fees from Eli Lilly & Company, and Novartis Pharmaceuticals. She is on the speaker's bureau for Merck and Company. SRC receives research support from Amgen, Pfizer, Novartis, Eli Lilly and Co. and consulting fees or honoraria from Eli Lilly and Co., Zelos, Merck and Co., Novartis, GlaxoSmithKline, Procter & Gamble, and Aventis. NK, JWW, EOT, LAM, MCH, TAH, and SBM had no conflicts.

## Authors' contributions

NK carried out the analysis and drafted the manuscript; JAC participated in the conceptual design, draft of the manuscript. SBM conceived the study and performed data acquisition; JWW participated in the analysis plan, and design of the study. EOT, LAM, SRC, MCH, TAH, participated in its design, coordination, and review of the study. All authors read and approved the final manuscript.
